# The Gray Mouse Lemur: A Model for Studies of Primate Metabolic Rate Depression

**DOI:** 10.1016/j.gpb.2015.06.001

**Published:** 2015-06-21

**Authors:** Kenneth B. Storey

**Affiliations:** Institute of Biochemistry and Department of Biology, Carleton University, Ottawa, ON K1S 5B6, Canada

The use of daily torpor and/or of multi-day torpor bouts during a hibernation season are energy-saving survival strategies that have been well-studied for many years, particularly for small mammals living in seasonally-cold environments. Both phenomena are characterized by a regulated suppression of metabolic rate, a slowing of many physiological processes (*e.g.*, heart and breathing rates, organ perfusion, kidney filtration, and neurological activity), and heterothermy that allows core body temperature (*T*_b_) to fall to near-ambient, often to values close to 0 °C for small hibernators [1–4]. All of these concepts are largely alien to our experience as humans. For example, hypothermia in humans is clinically diagnosed if core *T*_b_ drops to 34–35 °C and the risk of cardiac arrest and death is very high if core *T*_b_ is less than about 28 °C [5]. The natural capacity for metabolic suppression in humans is quite limited with only small reductions in metabolic rate and *T*_b_, when recorded in deep meditative states [6], during starvation [7], or in newborns exposed to hypoxia [8]. However, biomedical researchers envision a range of applications that can be derived from a thorough understanding of the natural mechanisms that regulate daily torpor and multi-day hibernation. Chief among these is the development of methods to expand the use of organ transplantation as a medical therapy by using preservation strategies derived from the natural mechanisms of torpor and/or hypothermia tolerance displayed by various mammalian species. Mechanisms to induce whole body torpor are also envisioned as a beneficial treatment when injured persons need to be transported long distances to medical care, when patient recovery could be aided by a prolonged torpid state, or even as an aid to long-term space flight. The best model animal for such studies has long been sought.

Long-standing models of torpor and hibernation are mainly rodents (*e.g.*, ground squirrels, marmots, hamsters, and mice), although considerable work has also been done on bats, some small marsupials and a few others [4]. Although much information about the molecular, biochemical and physiological mechanisms of natural hibernation has been derived from these studies [1–4], there are some questions about the applicability of this work to development of inducible torpor in humans. For example: (1) would the genes, proteins, and molecular mechanisms involved in torpor/hibernation in rodents also apply to humans? and (2) is endurance of very low *T*_b_ values, such as typically occurring during rodent hibernation, actually necessary for the preservation applications envisioned for humans or their isolated organs? The latter question is actually relevant in that some recent studies are showing that warm perfusion of excised human organs during transfer from donor to recipient is an effective and potentially less injurious option than the cold ischemia (packing in ice) that has been the standard for many years [9,10].

We believe that the ultimate model of relevance to human biomedical concerns would be a primate species that exhibits natural torpor/hibernation. Such species exist; studies in recent years have documented both daily torpor and seasonal hibernation among several Afrotropical mammals [11] including seven small lemur species from Madagascar, all belonging to the Cheirogaleidae family. Five of these are known to use both daily torpor and multi-day hibernation: the mouse lemurs (*Microcebus murinus* and *M. griseorufus*) and the dwarf lemurs (*Cheirogaleus medius*, *C. major*, and *C. crossleyi*) [12,13]. With the capacity for torpor/hibernation being present in these primate species, there is scope to predict that this ability may also be hidden within the human genome, and could potentially be awakened to aid development of inducible torpor as an aid to human tissue and organ preservation.

This optimal model for studies of primate torpor is the gray mouse lemur (*M. murinus*) (Figure 1), a small (weighing 60–110 g) nocturnal primate found mainly along the western coast of Madagascar and mostly in regions of lowland tropical dry forest, sub-arid thorn scrub, and spiny forest. Food is plentiful in the wet summer season when mean monthly maximum and minimum environmental temperatures are about 32 and 23 °C, respectively. However, the dry winter season is characterized by limited food availability and cool temperatures (mean monthly maximum and minimum are about 28 and 14 °C, but temperatures as low as ∼5 °C have been reported), setting up environmental conditions (low water, low food, and low temperature) that strain the energy budget of these small mammals. As a result, mouse lemurs enter torpor while sleeping and can also undergo multi-day hibernation [14,15]. Environmental factors including photoperiod, ambient temperature, and food availability shape the seasonal rhythms of these animals that include a winter resting period, an active summer breeding season, and an autumn fattening stage when lipid depots are laid down to fuel winter hypometabolism [14,16].

A large breeding colony of *M. murinus* has been maintained in Brunoy, France for more than 40 years and the animals have been used as a non-human primate model for many types of studies. In particular, as a relatively long-lived species (for its body mass), mouse lemurs are extensively studied as a primate model to understand the aging process, age-associated pathologies (*e.g.*, Alzheimer’s disease), and factors that could help to delay aging, such as calorie restriction [17–19]. This work has produced valuable information on age-dependent changes in endocrine systems, biological rhythms, thermoregulation, and sensory, cerebral, and cognitive functions.

Research in my laboratory centers on hypometabolism with a major focus on elucidating and exploring the biochemical mechanisms that regulate metabolic rate depression, including studies of gene and protein expression, enzyme regulation, posttranslational modification, signal transduction, microRNA control of mRNA transcripts, antioxidant defenses, chaperones, and intermediary energy metabolism [1,2,20–22]. Using both vertebrate and invertebrate models, we have been studying multiple formats of hypometabolism, *e.g.*, hibernation in cold climates, estivation in hot arid environments, anoxic tolerance, and life in a frozen state. As a result, we have identified conserved metabolic mechanisms that are used across the animal kingdom to coordinate and regulate metabolic rate depression, reprioritize ATP expenditures to conserve energy, stabilize macromolecules, and maximally extend viability in the hypometabolic state.

Based on these wide-ranging studies of metabolic regulation in diverse systems of hypometabolism, we have been able to discern the central features and regulatory requirements of hypometabolism across the animal kingdom. These include factors such as altered patterns of intracellular signaling by protein kinase cascades [*e.g.*, insulin signaling, AMP-dependent protein kinase (AMPK), glycogen synthase kinase 3 (GSK3), and mitogen-activated protein kinase (MAPK) pathways], coordinated regulation of multiple enzymes and proteins via reversible protein phosphorylation to suppress both anabolic functions (*e.g.*, protein synthesis, cell cycle, carbohydrate use for biosynthesis, and expression of many genes) and energy-expensive activities (*e.g.*, ion motive ATPases, thermogenesis), as well as selective gene/protein expression to produce cytoprotective proteins (*e.g.*, chaperones and antioxidants). From this, we have designed what we call a “toolkit” approach to evaluate the crucial molecular signatures of hypometabolism [23,24], signatures that have been identified by our group and others as integral to mammalian hibernation as well as other forms of environmental stress-responsive metabolic rate depression. Significantly, these signatures can now be assembled in multiplex assay formats (*e.g.*, 96-well microplates) that permit rapid, consistent, and sensitive analyses allowing multiple tissues and multiple experimental conditions to be compared on a single plate using technologies of PCR, ELISA, or Luminex. These technologies are particularly well-suited for making maximal use of very small tissue samples.

In the group of papers that follows, we put our toolkit of metabolic markers and our multiplex assay methods to use for a broad-based and comprehensive analysis of the biochemical responses to daily torpor in six organs of gray mouse lemurs. The studies include analysis of torpor-responsive changes in cell signaling pathways (insulin-signaling Akt/PI3K pathway, MAPKs, AMPK, and GSK3) [25–27], mRNA expression changes for selected genes that were previously linked with the hibernation phenotype in ground squirrels [28], controls on transcription (histone modification) and translation (mTOR and ribosomal proteins) [26,27], carbohydrate fuel regulation at the pyruvate dehydrogenase locus [27], and cytoprotective mechanisms (antioxidant defenses and heat shock proteins) [29]. A final study examines immune responses to torpor in the lemur intestine [30]. Overall, this group of studies illustrates the conservation during lemur torpor of many of the basic regulatory parameters of metabolic rate depression that are found across phylogeny, demonstrates the power of a multiplex approach to biochemical analysis, and illustrates some new features of torpor such as cytokine responses by the immune system in intestine. This validates the use of the lemur model for exploring both the characteristics of warm temperature torpor in a primate and sets the stage for in-depth studies of the genomics and proteomics of lemur torpor that will lead to identification of the critical elements of torpor induction and control that could be applied to improve human organ preservation.

## Author’s contributions

KBS wrote and edited this manuscript.

## Competing interests

The author has declared no competing interests.

## Figures and Tables

**Figure 1:**
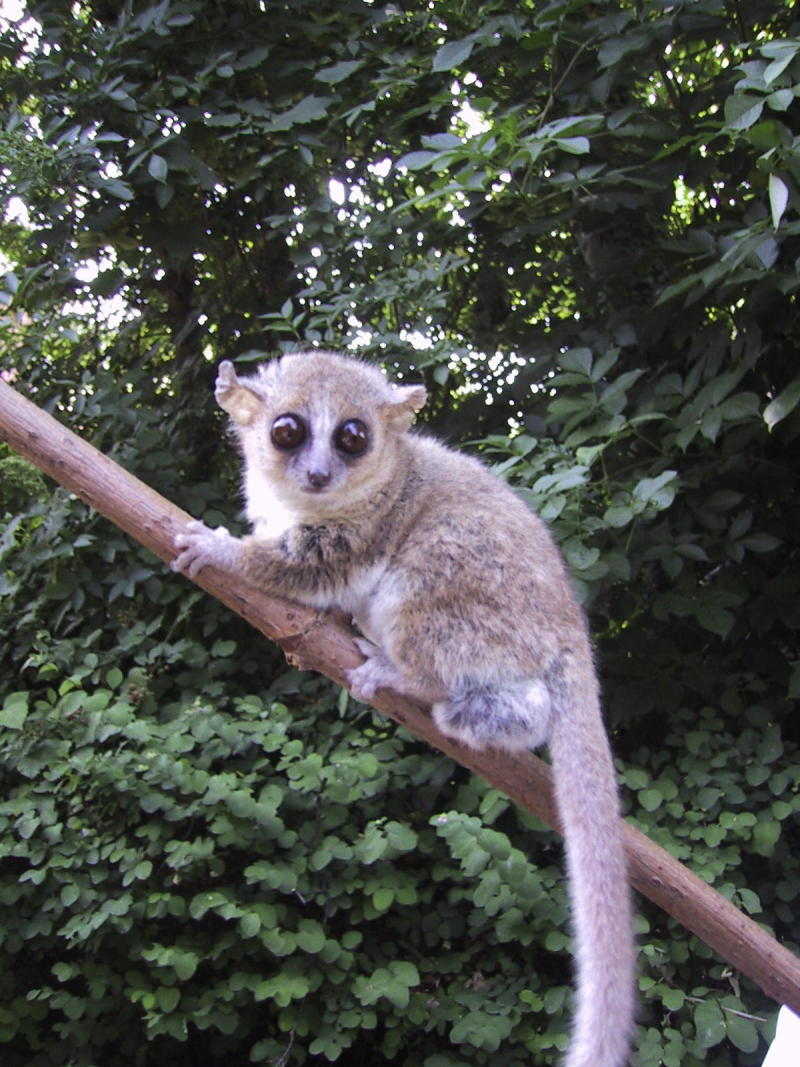
**Photo of a gray mouse lemur (*Microcebus murinus*) in nature** Used with permission of Martine Perret, Muséum National d′Histoire Naturelle, Brunoy, France.
